# Anticancer potential of *Bacillus coagulans* MZY531 on mouse H22 hepatocellular carcinoma cells via anti-proliferation and apoptosis induction

**DOI:** 10.1186/s12906-023-04120-7

**Published:** 2023-09-13

**Authors:** Zhongwei Zhao, Qian Yang, Tingting Zhou, Chunhong Liu, Manqing Sun, Xinmu Cui, Xuewu Zhang

**Affiliations:** 1 Medical College, Yanbian University, Yanji, 133002 Jilin Province P.R. China; 2 College of Special Education, Changchun University, Changchun, 130022 P.R. China; 3 Innovation Practice Center, The Changchun University of Traditional Chinese Medicine, Changchun, 130000 P.R. China

**Keywords:** Probiotic, *Bacillus coagulans*, Apoptosis, Anticancer

## Abstract

*Bacillus coagulans* have recently revealed its anticancer effects, but few investigations are available on their effects on liver cancer proliferation, and the precise mechanism to mark its impact on apoptosis-related signaling pathways has yet to be elucidated. The aim of this study was to evaluate the anti-proliferative effect of *B. coagulans* MZY531 and apoptosis induction in the mouse H22 hepatocellular carcinoma cell line. The anti-proliferative activity of *B. coagulans* MZY531 was evaluated by Cell Counting Kit-8 (CCK-8) assay, and cell apoptosis was revealed with Terminal Deoxynucleotidyl Transferase (TDT)-mediated dUTP Nick-End Labeling (TUNEL) staining and flow cytometric analysis. The expressions of apoptosis-related protein were determined by western blot analysis. The CCK-8 assay revealed that *B. coagulans* MZY531 inhibited the H22 cells proliferation in a concentration-dependent manner. TUNEL staining revealed an increased apoptosis rate in H22 cells following intervention with *B. coagulans* MZY531. Furthermore, flow cytometric analysis showed that *B. coagulans* MZY531 treatment (MOI = 50 and 100) significantly alleviated the H22 cells apoptosis compared with the control group. Western blot analysis found *B. coagulans* MZY531 significantly decreased level of phospho-PI3K (p-PI3K), phospho-AKT (p-AKT), and phospho-mTOR (p-mTOR) compared with the control group. Furthermore, H22 cells treatment with *B. coagulans* MZY531 enhanced the expression of caspase-3 and Bax and jeopardized the expression of Bcl-2. Taken together, apoptosis induction and cell proliferation inhibition via PI3K/AKT/mTOR and Bax/Bcl-2/Caspase-3 pathway are promising evidence to support *B. coagulans* MZY531 as a potential therapeutic agent for cancer.

**Supplementary Information:**

The online version contains supplementary material available at 10.1186/s12906-023-04120-7.

## Introduction

Probiotics are microecological regulator that provides health benefits to the host [1, 2]. It can improve the intestinal microecological environment, enhance immune function, regulate glucose and lipid metabolism, and play a pivotal role in anti-inflammatory responses, anti-oxidation, etc. Probiotics can interact with the host, elicit health benefits and maintain homeostasis in the host [3]. Previous studies have found that some lactic acid bacteria and their metabolites have inhibitory effects on multiple malignant tumors, including colon and stomach cancer [4, 5], breast cancer [6], liver cancer [7, 8], and lung cancer [9]. A growing number of studies reported probiotics playing an antitumor role by inhibiting tumor cell proliferation, producing anticancer metabolites, adjusting intestinal flora, and activating immune function [10–12]. Probiotics have revealed their therapeutic benefits by inducing tumor cell apoptosis. For instance, probiotics induce mitochondrial apoptosis in colorectal cancer cells [13]. *Lactobacillus casei* and *L. paracasei* inhibit cancer by regulating the expression of Bcl-2 and caspase family proteins [14]. A recent study showed that *L. gasseri* inhibits the PI3K/AKT signaling pathway, reduces cancer cell survival, and induces apoptosis [15]. Therefore, inducing tumor cell apoptosis may be one of the important anticancer ways for probiotics, however, the mechanism needs further evaluation.

*Bacillus coagulan* is a spore-forming Gram-positive bacteria that can produce lactic acid. Based on the safety and extensive applications of *B. coagulans*, the Chinese Health Commission approved their use in common food. *B. coagulans* has garnered the attention of many researchers as one of the most common and significant probiotics in people’s everyday lives [16]. The bacteria also produce spores in addition to lactic acid at favorable temperatures and pH. The bacteria are resistant to high temperatures. They can grow at pH values in the stomach and intestines. *B. coagulans* convey health benefits by resisting oxidants, maintaining normal digestive tract flora, preventing enteritis, and regulating the immune response [17, 18]. Previous studies demonstrated that *B. coagulans* have an antitumor role in vitro and mouse models. For example, Madempudi and Kalle found that the heat-killed filter stabilized culture supernatant (hsup) of *B. coagulans* induces apoptosis by regulating Bax/Bcl-2 ratio, reducing Mitochondrial Membrane Potential (MMP), releasing cytochrome c, activating Caspase 3 and cleaving PARP [19].

The exact mechanism to probe the anticancer activity of *B. coagulans* is not clear. Some of the proposed mechanisms may affect the metabolism, immune, and protective functions of colon and may also stimulate the apoptosis of tumor cells [20]. However, to our knowledge, no published study has reported the toxicity and antitumor effects of *B. coagulans* in H22 hepatoma cells. Therefore, our preliminary work aims to explore the impact and mechanism of *B. coagulans* in the proliferation and apoptosis of H22 hepatoma cells and to lay a theoretical foundation for their application. In this study, *B. coagulans* MZY531 is a potential probiotic strain, which is isolated from traditional fermented food in China. It displays some probiotic characteristics based on in vitro and in vivo studies, including viability at low pH, tolerance to bile salts and antibiotic susceptibility. However, the anticancer activity of *B. coagulans* MZY531 against liver cancer is still unknown. The anti-proliferative activity of *B. coagulans* MZY531 for H22 hepatoma cells was evaluated by CCK-8 assay. The induction of apoptosis was analyzed via TUNEL staining and flow cytometry. The molecular mechanism of apoptosis was analyzed via western blotting analysis.

## Materials and methods

### Strain and medium

*B. coagulans* MZY531 was provided by Jilin Mingzhiyuan Biotechnology Co. Ltd. The strain was preserved in China’s Typical Culture Collection Center with the preservation number CCTCC No.2,021,662.The living *B. coagulans* MZY531 was inoculated in liquid GPY medium, incubated at 50 ℃ in a shaking table at 180 rpm for 20 h, and centrifuged (3000 r/min, 4 ℃, 10 min). The bacterial suspension was prepared with sterile normal saline, and the number of viable bacteria was adjusted to 1 × 10^9^ Colony Forming Units (CFU) / mL and stored at 4 ℃ for standby.

### Growth curve of *B. coagulans* MZY531

5% activated bacterial liquid was inoculated in 500 ml fermentation medium, taking 5 ml every 2 h, and determine the pH value with a pH meter (sartorius PB-10, Germany). The bacterial liquid was diluted, spread on GPY agar plate, incubated for 48 h at 50 ℃, and counted the colonies.

### Cell culture

The mouse H22 hepatocellular carcinoma cells were purchased from Jiangsu KeyGEN BioTech Co., Ltd. The cells were cultured in growth medium RPMI1640 (Jiangsu KeyGEN Biotech Corp., Ltd.) containing 10% FBS (Cyagen Biosciences Lnc.) and 1% penicillin/streptomycin (Beijing Kulaibo Technology Co., Ltd.) followed by incubation with 95% relative humidity and 5% CO_2_ at 37 ℃. The culture medium was changed every 1–2 days. The supernatant culture media was collected after the cell monolayer had covered 80% surface of the cell culture plate, and the bottom was then cleaned with PBS (Wuhan Servicebio Technology Co., Ltd.). The bottom cells were digested with trypsin (Beijing Kulaibo Technology Co., Ltd.), and the collected liquid was used for subculture or experiments. The cells in a logarithmic phase were used in all experiments.

### Cell viability assay

The Cell Counting Kit-8 (U.S. Everbright lnc., Jiangsu, China) was used to assess the proliferation and viability of H22 cells in *B. coagulans* MZY531 cell suspension [21]. 1 × 10^4^ H22 cells were inoculated into 96 well culture plates and added with *B. coagulans* MZY531 (MOI = 0, 1, 10, 50, 100) for 24 h. 5-Fluorouracil (5-FU) (100 µg/ml) purchased from Shanghai yuanye Bio-Technology Co., Ltd (Shanghai, China) was used as a positive control. CCK-8 solution with a volume ratio of 10% (V/ V) was added and incubated for 3 h. The SPECTROMAX ABS plus (Molecular Devices, Shanghai, China) read the absorbance at 450 nm.

$$Cell{\rm{ }}\ viability\% {\rm{ }} = {A_{test}}/{A_{control}} \times 100\% $$

### TUNEL staining

The TUNEL staining was employed to study the effects of *B. coagulans* MZY531 on H22 Cells [22]. 2 × 10^5^ H22 cells were plated in each hole of a 6-well plate with sterile coverslips and were incubated with *B. coagulans* MZY531 (MOI = 0, 50, 100) for 24 h. After growing to an appropriate size, the cells were washed thrice with PBS and later fixed with 4% paraformaldehyde for 30 min. The cells were washed with PBS after fixation, incubated with 100 µL 0.3% Triton X-100 at room temperature for 20 min, and rewashed with PBS 3 times. The cells were covered with buffer in TUNEL kit (Wuhan Servicebio Technology Co., Ltd.) and incubated at room temperature for 10 min. The cells were covered with the mixture of TDT enzyme: dUTP: buffer (1:5:50) and incubated in a 37 ℃ incubator for 2 h. PBS was used to wash the cells 3 times (5 min each time). The PBS was removed, and 4, 6-diamidino-2-phenylindole (DAPI) dye was used for staining. The samples were incubated in dark at room temperature for 10 min. The images were collected and observed under a fluorescence microscope (DAPI ultraviolet excitation wavelength 330–380 nm, emission wavelength 420 nm, blue light; Cy3 excitation wavelength 510–560 nm, emission wavelength 590 nm, red light; DAPI stained cell nucleus was blue under ultraviolet excitation, Cy3 fluorescein-labeled apoptotic cells and the nucleus was red). The number of TUNEL-positive cells was counted by Image J software.

### Flow cytometry

2 × 10^5^ H22 cells were inoculated into a 6-well plate [23]. After intervention with *B. coagulans* MZY531 (MOI = 0, 50, 100) and 5-FU (100 µg/mL) for 24 h, the cells were digested with trypsin without EDTA and centrifuged (4 ℃, 1000×g, 2 min). 1 × 10^5^ H22 cells were suspended with 100 µL 1×combined buffer, stained with Annexin V/(Propidium Iodide) PI solution, and incubated at 37 ℃ for 15 min. Flow cytometry was used to analyze the fluorescence emission spectra of Y®488 Annexin V excited by 488 nm laser at 530 nm (FITC channel) and 617 nm (PI channel).

### Protein hybridization analysis

H22 cells (3 × 10^5^cells/hole) in a logarithmic growth phase were plated in a 6-well plate and were incubated with *B. coagulans* MZY531 (MOI = 0, 50, 100) and 5-FU (100 µg/mL). After 24 h, the whole cell extract was prepared with RIPA buffer containing 1 mm PMSF. The protein concentration was detected by the BCA method, separated by SDS-PAGE electrophoresis, and transferred to the PVDF membrane. Western blot analysis was performed using the following primary anti-rabbit monoclonal antibodies: β-actin (bsm-52846R, BIOSS), PI3K (bs-10657R, BIOSS), p-PI3K (bs-5570R, BIOSS), AKT (bs-0115R, BIOSS), p-AKT (bs-0876R, BIOSS), mTOR (bsm-54471R, BIOSS), p-mTOR (bs-5331R, BIOSS), and Bax (GTX109683, GeneTex), caspase-3 (GTX110543, GeneTex) and Bcl-2 (GTX100064, GeneTex). The membrane was incubated with anti-rabbit second antibody conjugated with horseradish peroxidase at 37 ℃ for 1 h. the strips were quantified using image quant Las 4000 (Fuji film, Tokyo, Japan), and β-actin was used as a loading control.

### Data processing

SPSS 22.0 was used for statistical analysis. Graphpad Prism 5 was used for drawing graphs, and the results were expressed as means ± SD. One-way ANOVA and Duncan multiple comparisons were used for multivariate comparison. P < 0.05 were regarded as statistically significant.

## Results

### Growth curve of *B. coagulans* MZY531

**Fig. 1:**
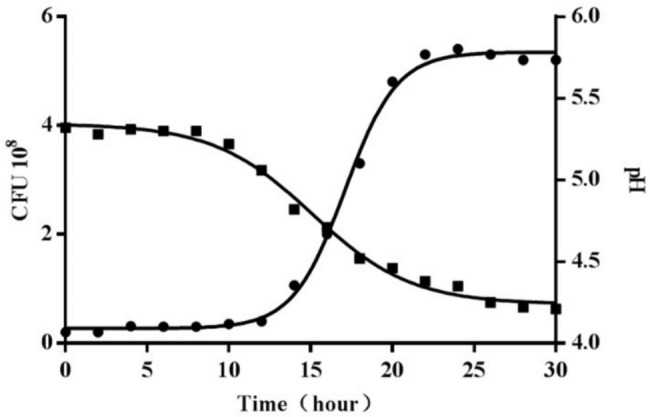
Growth and acid production rate curve of *B. coagulans* MZY531. The number of viable bacteria and pH value of *B. coagulans* MZY531 at different times were detected by flat colony counting method and pH meter, respectively

The *B. coagulans* MZY531 fermentation broth pH decreased with incubation and tended to be stable at 30 h (pH = 4.2). The strain exhibited mildish growth in 0–14 h culture period and logarithmic growth rate after 15 h. After 25 h, the growth rate decreased, and the number of viable bacteria reached the maximum of 5 × 10^8^ CFU/mL, after which the strain grew into a stable phase (Fig. 1).

### ***B. coagulans*** MZY531 decreased mouse H22 hepatocellular carcinoma cells viability

**Fig. 2:**
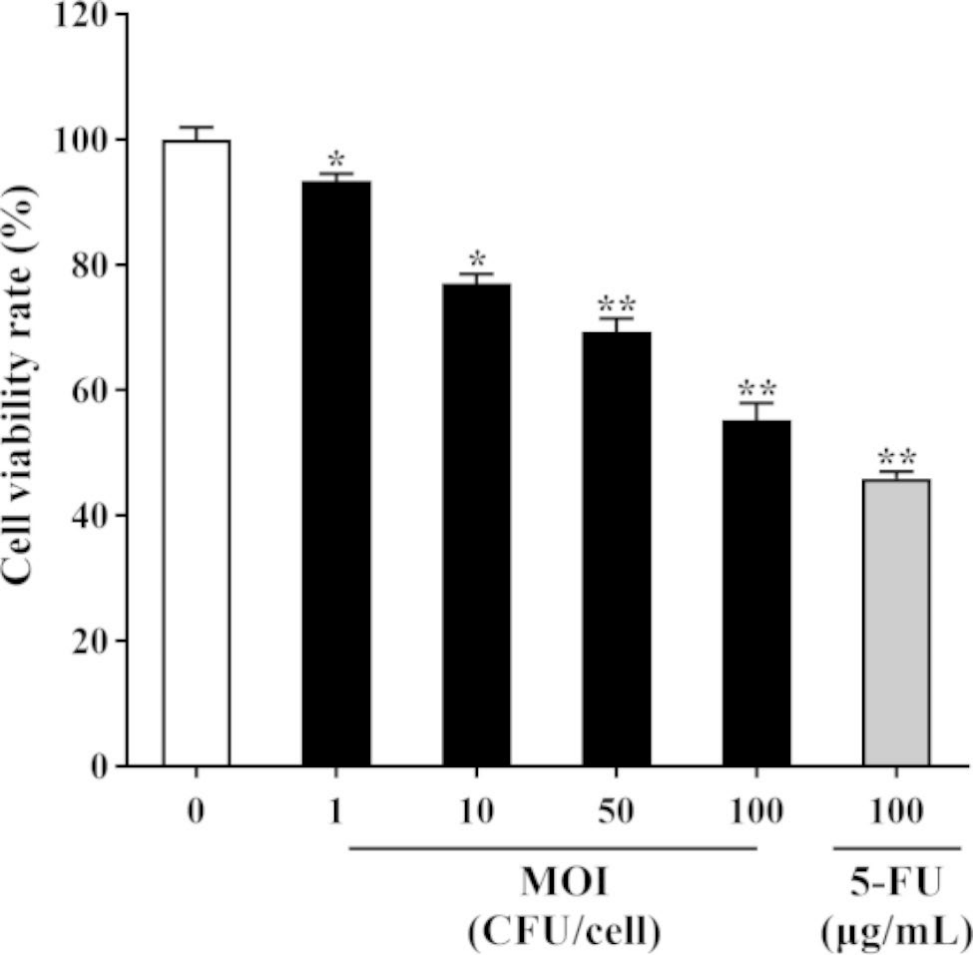
Effect of *B. coagulans* MZY531 on the mouse H22 hepatocellular carcinoma cells viability. Cell viability was determined by the CCK-8 assay after treatment with various concentrations of *B. coagulans* MZY531 (MOI = 0, 1, 10, 50, 100) for 24 h. Data were presented as means  ± SD of triplicates. Data are analyzed by one-way ANOVA and Tukey post doc assay using SPSS software. *P < 0.05, **P < 0.01 vs. control group

The inhibitory effect of *B. coagulans* MZY531 on mouse H22 hepatocellular carcinoma cells is shown in Fig. 2. The findings revealed that *B. coagulans* MZY531 treatment significantly hampered the growth of mouse H22 hepatocellular carcinoma cells. *B. coagulans* MZY531 inhibited the proliferation of H22 hepatoma cells in a concentration-dependent manner. With the increase of *B. coagulans* MZY531 concentration, the viability of H22 cells decreased gradually. After the bacteria with the concentration of MOI = 100 treated H22 cells for 24 h, the inhibition rate reached 47.26 ± 1.86%, significantly higher than that of the blank control group. The IC_50_ value of *B. coagulans* MZY531 is MOI = 107.

### ***B. coagulans*** MZY531 induced H22 hepatoma cells apoptosis

**Fig. 3:**
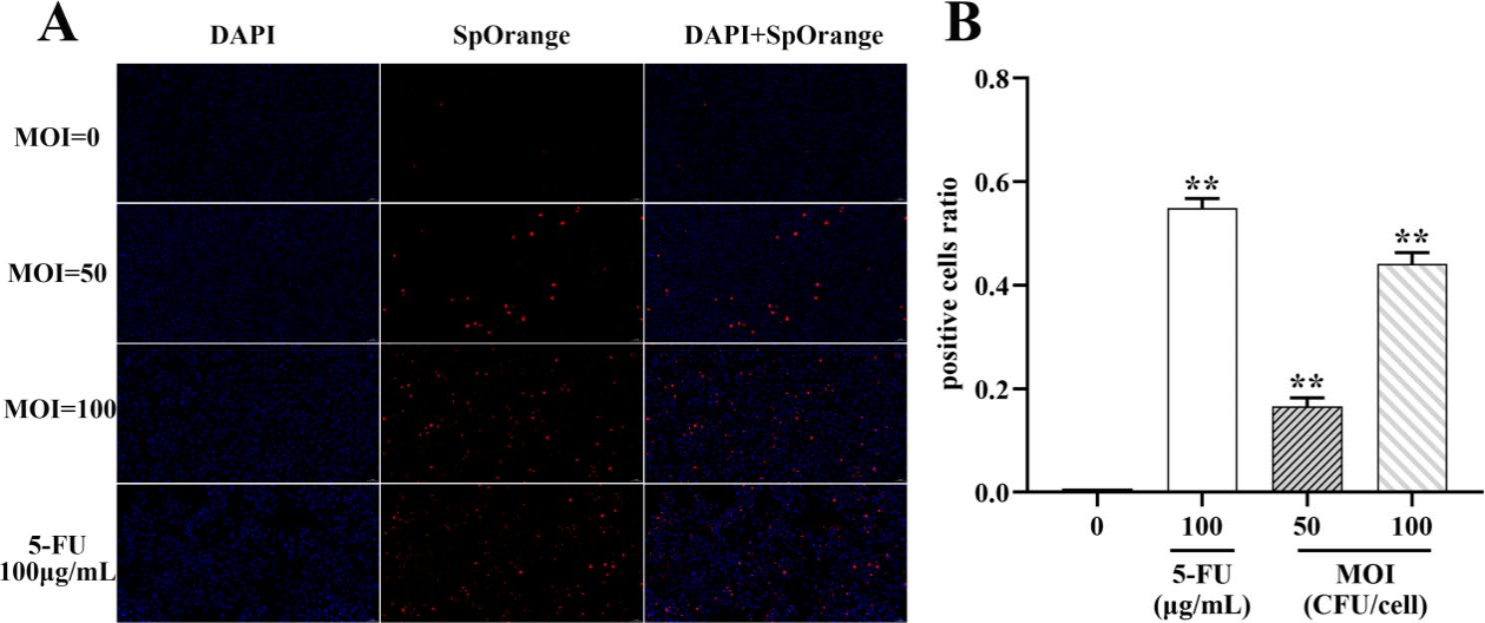
Effect of *B. coagulans* MZY531 on apoptosis rate of H22 cells. (**A**) TUNEL staining to determine cell apoptosis in mouse H22 hepatocellular carcinoma cells in each group under 20× visual field. (**B**) Quantitative analysis of TUNEL staining in each group. Data were presented as means ± SD of triplicates. Data are analyzed by one-way ANOVA and Tukey post doc assay using SPSS software. **P < 0.05 compared with control group

TUNEL staining revealed that *B. coagulans* MZY531 (MOI = 0, 50 and 100) induced apoptosis in H22 (Fig. 3). the findings revealed that the increase of MOI decreased the number of normal H22 hepatoma cells stained blue. It increased the number of apoptotic cells stained red increased. Apoptosis was triggered in H22 hepatoma cells by *B. coagulans* MZY531, with a higher apoptotic degree than the positive group recorded at MOI = 100. The apoptotic degree of 100 µg/mL 5-FU was nearly 50%.

### *B. coagulans* MZY531 increased early versus late apoptotic cells

**Fig. 4:**
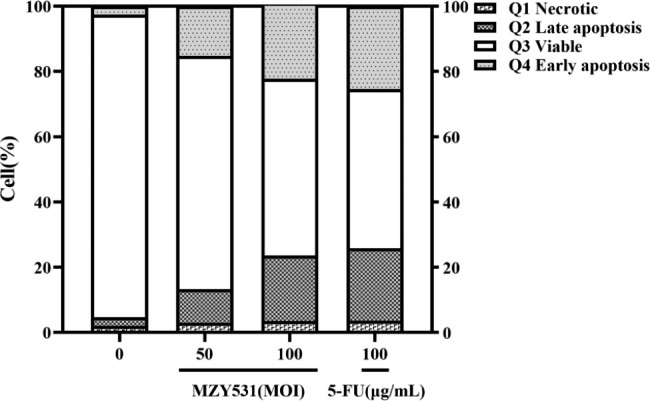
Effect of *B. coagulans* MZY531 on apoptosis cycle of H22 cells. *B. coagulans* MZY531 were incubated in H22 cells of different MOI (0, 50, and 100) and 5-FU (100 µg/mL) for 24 h. Q1–Q4 represents necrotic cells, late apoptotic cells, viable cells, and early apoptotic cells

Flow cytometry showed that *B. coagulans* MZY531 could induce apoptosis of H22 cells. The apoptosis rates of H22 cells treated with *B. coagulans* MZY531 (MOI = 50,100) for 24 h were 25.53% and 42.39%, significantly higher than the control group (20.19%, 37.05%) (Fig. 4).

### ***B. coagulans*** **MZY531 regulated the apoptosis of mouse H22 hepatocellular carcinoma cells by the PI3K/AKT/mTOR signaling pathway**

**Fig. 5:**
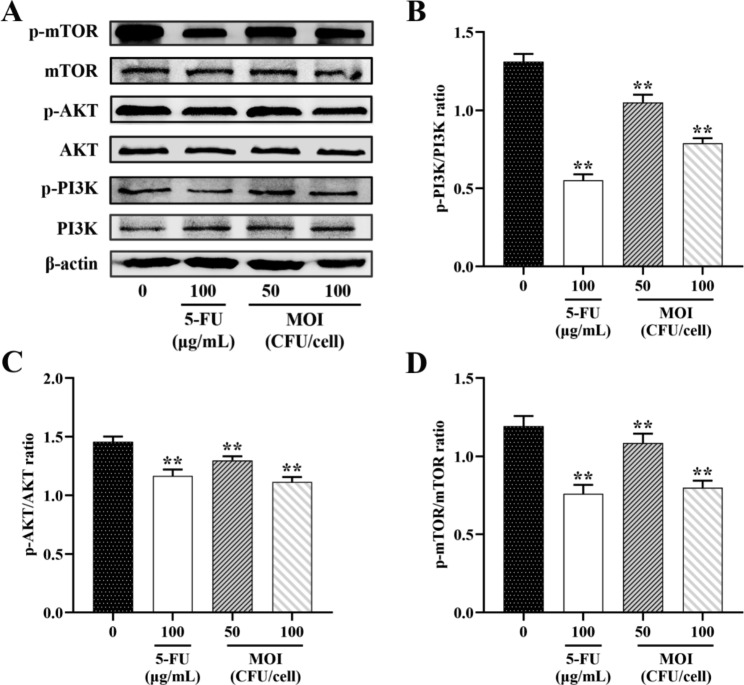
(**A**) Effect of *B. coagulans* MZY531 on the expression of PI3K, AKT, mTOR. The expression of Bax (**B**), Bcl-2 (**C**) and Caspase-3 (**D**) protein was detected by western blot analysis. The data were analyzed by one-way ANOVA: **P < 0.01 *vs.* control group, means ± SD. β-actin was used as standard control

To determine the signaling pathway activated by *B. coagulans* MZY531 in H22 hepatocellular carcinoma cells, we detected the expression of PI3K/AKT/mTOR pathway-related proteins. The *B. coagulans* MZY531-treated group showed a significant reduction in the expression of phosphorylated proteins PI3K, AKT, and mTOR compared to the control group (Fig. 5A). H22 cells PI3K and AKT phosphorylation levels were similarly shown to be reduced by *B. coagulans* MZY531 (Fig. 5B-D). These results indicated that *B. coagulans* MZY531 could regulate the apoptosis of H22 hepatoma cells by downregulating the PI3K/AKT/mTOR signaling pathway.

### ***B. coagulans*** MZY531 promote the apoptosis of H22 hepatoma cells by Bax/Bcl-2/ caspase-3 signal pathway

**Fig. 6:**
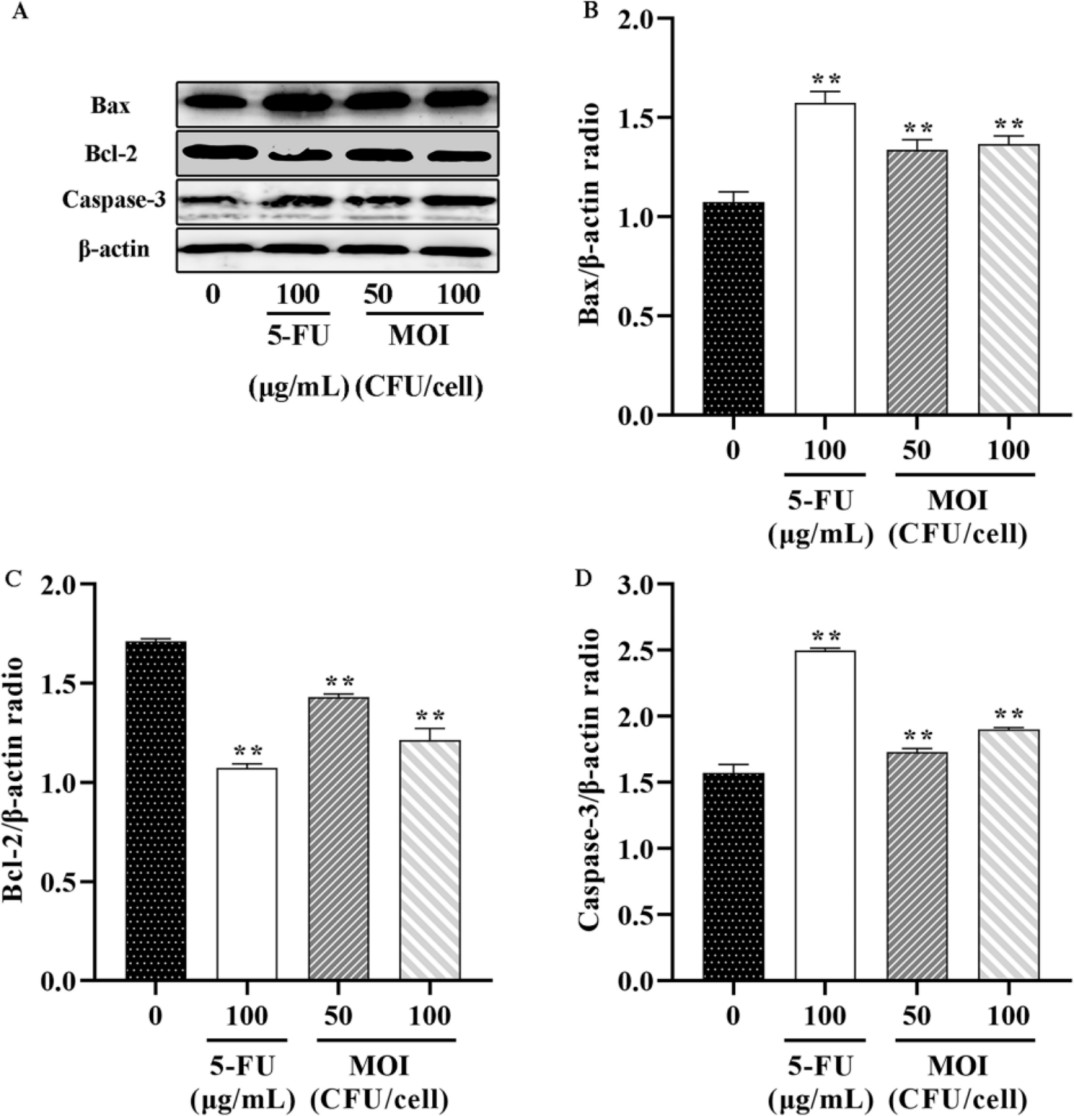
Effects of *B. coagulans* MZY531 on the protein levels of Bax, Bcl-2 and Caspase-3 in H22 cells. (**A**) representative pictures of western blots demonstrating the levels of Bax, Bcl-2 and Caspase-3. (**B**) (**C**) (**D**) Histograms summarizing the results presented in a data are presented as the means ± SD of three independent experiments. *P < 0.05 and **P < 0.01 *vs.* control group

The changes in total protein and activated protein in the apoptotic signal pathway were detected. We studied the possible mechanism of apoptosis of H22 hepatoma cells induced by *B. coagulans* MZY531. The results showed that *B. coagulans* MZY531 upregulated the expression of Caspase-3 and Bax and significantly inhibited Bcl-2 (Fig. 6). In addition, a low concentration of *B. coagulans* MZY531 can promote the apoptosis of H22 hepatoma cells, but the effect is not particularly apparent. Moreover, the increase of *B. coagulans* MZY531 concentration could effectively induce H22 cells apoptosis, which showed a significant concentration-effect relationship.

## Discussion

The induction of tumor cell apoptosis has been revealed as a pronounced approach for the prevention and treatment of cancer and is the key indicator for screening and evaluating the efficacy of anticancer drugs [24, 25]. The current preliminary study reported that *B. coagulans* MZY531 impedes the growth and apoptosis of H22 hepatoma cells. The results showed that *B. coagulans* MZY531 can inhibit the growth of hepatoma cells. The current study found that the PI3K/AKT/mTOR and the caspase-3/Bax/Bcl-2 signaling pathways involved *B. coagulans* MZY531 mediated inhibition of liver cancer progression. These results suggest that *B. coagulans* MZY531 can inhibit the proliferation and induce apoptosis of H22 hepatoma cells.

The CCK-8 assay was used to determine whether *B. coagulans* MZY531 could decrease mouse H22 hepatocellular carcinoma cells viability. Increased *B. coagulans* MZY531 concentration significantly reduces H22 cells viability. In addition, TUNEL staining was performed to identify apoptosis as the cause of cell death in H22 hepatoma cells treated with *B. coagulans* MZY531. The increase in *B. coagulans* MZY531 concentration significantly increased the apoptotic cells in H22 hepatoma cells. The cell volume became smaller with irregular morphology. Other studies have also confirmed that the death of tumor cells caused by probiotics is caused by apoptosis rather than necrosis [23, 26, 27]. Our flow cytometry results further demonstrated that *B. coagulans* MZY531 induced the decrease of H22 hepatoma cells activity, which was caused by apoptosis.

A complex network controls apoptosis, and the PI3K/AKT/mTOR signal transduction pathway is an important pathway to regulate cell apoptosis, which regulates cell growth, survival, and migration in the process of cancer progression and metastasis [28]. PI3K activates AKT, which in turn causes mTOR phosphorylation through a large number of regulators. A series of upstream or bypass signaling molecules activate the PI3K/AKT/mTOR. It directly phosphorylates apoptosis-related proteins or indirectly changes the gene expression level of apoptosis-related proteins, thus playing a key role in inhibiting apoptosis and promoting cell proliferation [29, 30]. Therefore, PI3K/AKT/mTOR pathway inhibition is essential in reducing cancer cell viability. Previous studies have shown that *L. rhamnosus* GG and its metabolites can inhibit cytokine-induced apoptosis of human or mouse intestinal epithelial cells by downregulating the p38/MAPK activation and upregulating the PI3K/AKT cascade [31]. *Bifidobacterium animalis* subsp *lactis* BI-04 can delay benzo [a] pyrene (BAP)-induced apoptosis of colon epithelial cells by upregulating the PI3K/AKT signaling pathway and downregulating p53 gene expression [32]. In this study, H22 hepatoma cells treated with *B. coagulans* MZY531 showed a concentration-dependent reduction in the expression of p-PI3K, p-AKT and p-mTOR compared to the control group. In addition, we also studied the effect of phosphorylated PI3K/AKT/mTOR protein expression and cell phenotype on response to *B. coagulans* MZY531 stimulation. The findings revealed that the protein levels of p-PI3K, p-AKT, and p-mTOR in H22 cells were significantly decreased after treatment with *B. coagulans* MZY531 for 24 h.

To sum up, *B. coagulans* MZY531 has an antitumor effect on H22 hepatoma cells, which can reduce cell viability and induce apoptosis by activating the PI3K/AKT/mTOR signaling pathway. Blocking the overactivated PI3K/AKT/mTOR signaling pathway may be a potential target of *B. coagulans* MZY531 in treating hepatocellular carcinoma because it regulates cell growth and proliferation. However, the mechanism needs to be further confirmed in relevant human models.

It is well-known that Bcl-2 family proteins play an important role in regulating apoptosis [33]. Bcl-2 is an apoptosis inhibitor, requiring high levels of Bcl-2 to maintain intracellular gene transfer and other necessary changes to block apoptosis. Bax is an apoptotic protein. Bax and Bcl-2 interact to regulate apoptosis and form a complex regulatory network. Therefore, the expression of Bax may need to be upregulated when the Bax level is low. In addition, caspase family proteases are downstream targets of Bax and Bcl-2 in the mitochondrial apoptosis signaling pathway. In particular, Caspase-3 plays a crucial role in the terminal and executive stages of apoptosis induced by various stimuli [34]. Interestingly, in this study, *B. coagulans* MZY531 showed its ability to restore the apoptosis pathway of H22 hepatoma cells. *B. coagulans* MZY531 can activate the pro-apoptotic factor, Bax, inhibit the anti-apoptotic protein Bcl-2 expression, activate caspase-3, and induce apoptosis. The results are in line with the previous commentaries on the same topic. Some lactic acid bacteria strains and their secretory components have anti-proliferation and pro-apoptotic effects on cancer cells by activating pre-caspases, downregulating anti-apoptotic protein Bcl-2, and upregulating pro-apoptotic protein Bax. For instance, the culture supernatant of three *L. rhamnosus* isolated from breast milk revealed good anticancer activity by regulating the expression of Bcl-2 family proteins and caspase family proteins in cancer cells [35]. *L. paracasei* K5 can induce apoptosis of human colon cancer Caco-2 cells in a time and concentration-dependent manner by regulating the expression of specific Bcl-2 family proteins [36]. The S-layer protein isolated from the surface of *L. acidophilus* CICC 6074 can promote the apoptosis of human colon cancer HT-29 cells through the mitochondrial pathway [37]. In addition, *B. coagulans* MZY531 promotes apoptosis by increasing the Bax level and decreasing Bcl-2 level, which is also related to the PI3K/AKT/mTOR pathway. The conclusion provides an upstream factor supplement for our experiment.

## Conclusion

The research indicates that *Bacillus coagulans* MZY531 demonstrates its anti-proliferative effects through multiple mechanisms, such as inducing apoptosis, arresting the cell cycle, and modulating critical signaling pathways crucial for cancer cell growth and survival. These findings offer valuable insights into the molecular mechanisms responsible for the inhibitory effects of *Bacillus coagulans* on H22 hepatoma cells. Moreover, as a probiotic bacterium, *Bacillus coagulans* is generally recognized as safe and well-tolerated, with minimal associated side effects. Consequently, the study’s outcomes establish a strong foundation for future investigations and hold significant promise for the development of innovative, safe, and efficient strategies in combating liver cancer.

### Electronic supplementary material

Below is the link to the electronic supplementary material.

Supplementary Material 1: Raw data of protein bands.

## Data Availability

All data generated or analysed during this study are included in this published article.
