# Supplementary material for: Anticancer potential of Bacillus coagulans MZY531 on mouse H22 hepatocellular carcinoma cells via anti-proliferation and apoptosis induction
Source: BMC Complement Med Ther. 2023 Sep 13;23:318. doi: 10.1186/s12906-023-04120-7 (PMC10498517; doi:10.1186/s12906-023-04120-7)

Bax-21kDa

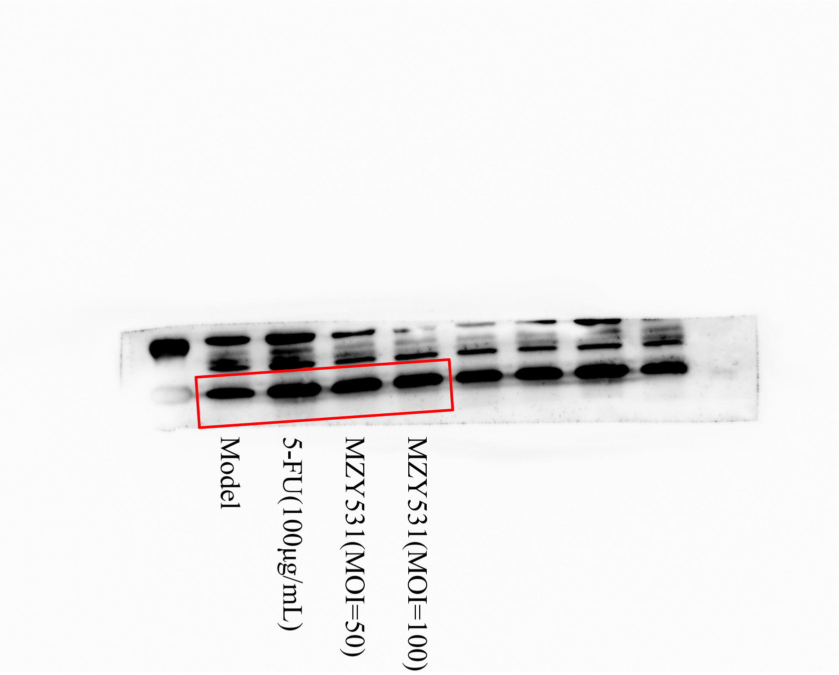

Bcl-2-26kDa

Repeat 1

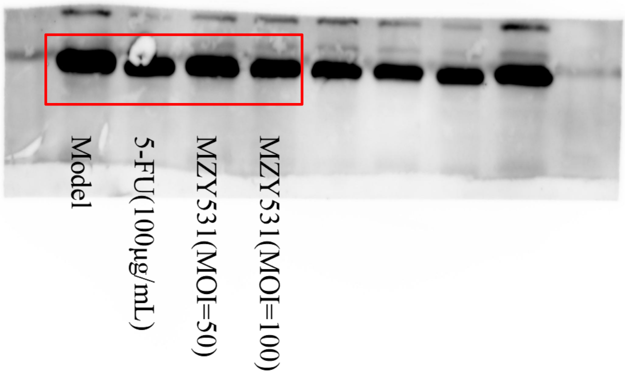

Caspase-3-32kDa

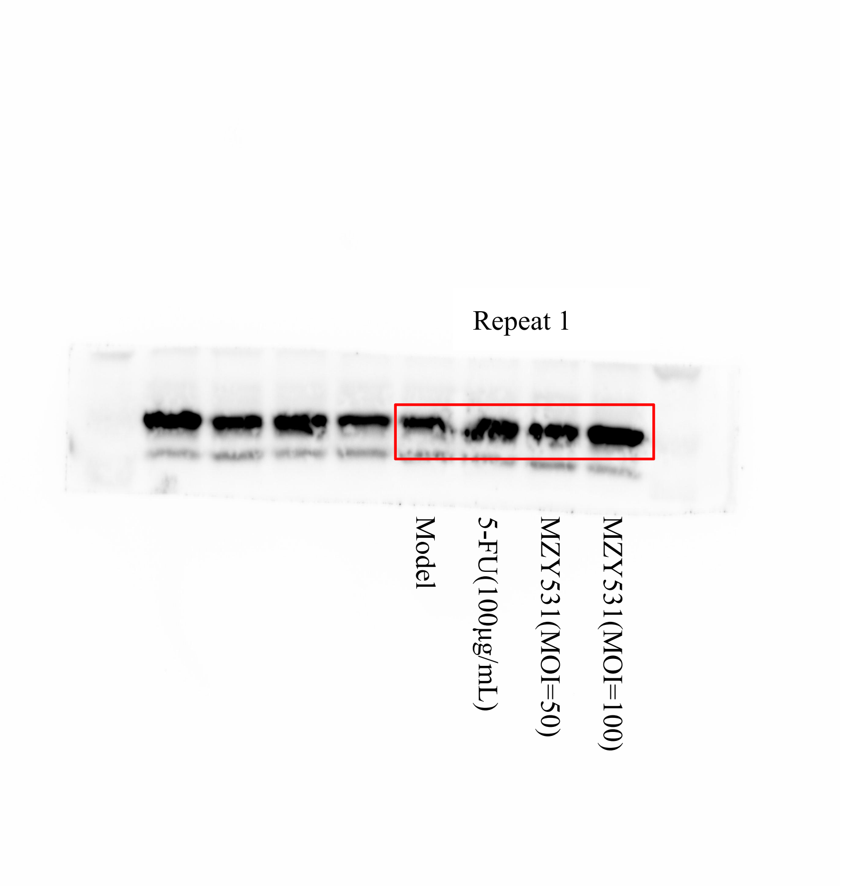

β-actin-42kDa

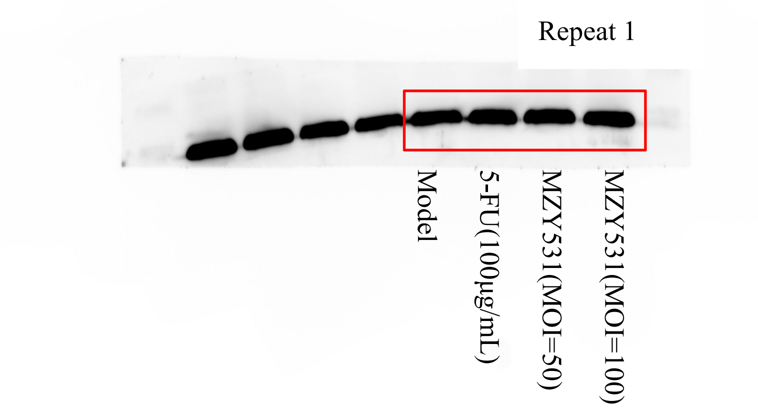

β-actin-42kDa

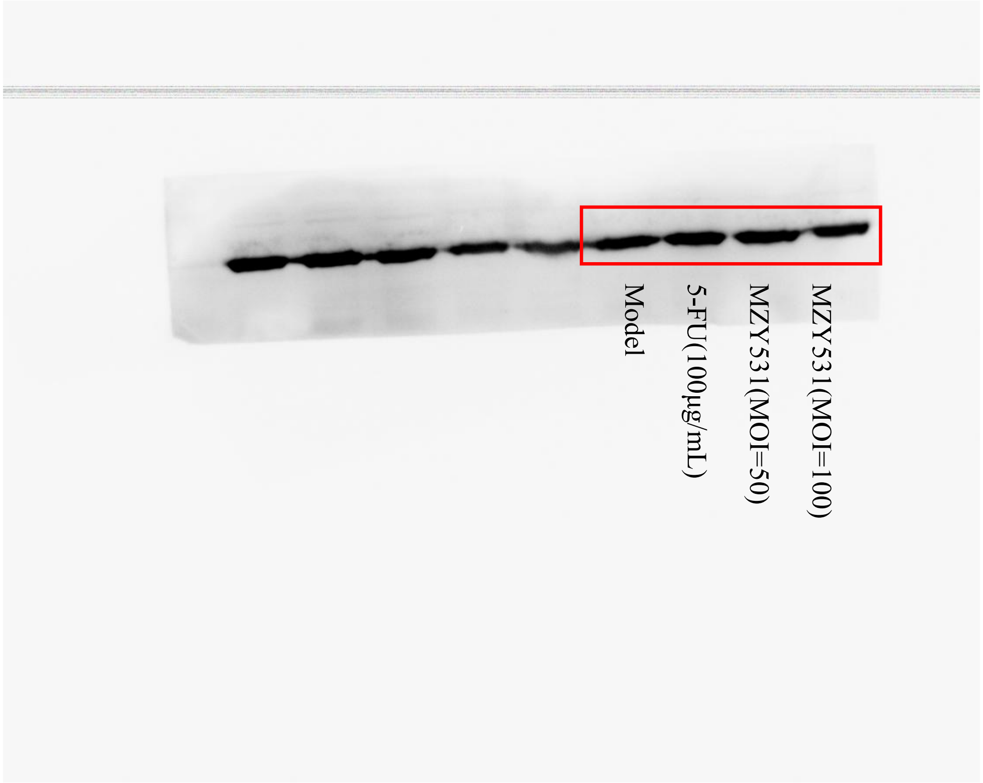

PI3K-123kDa

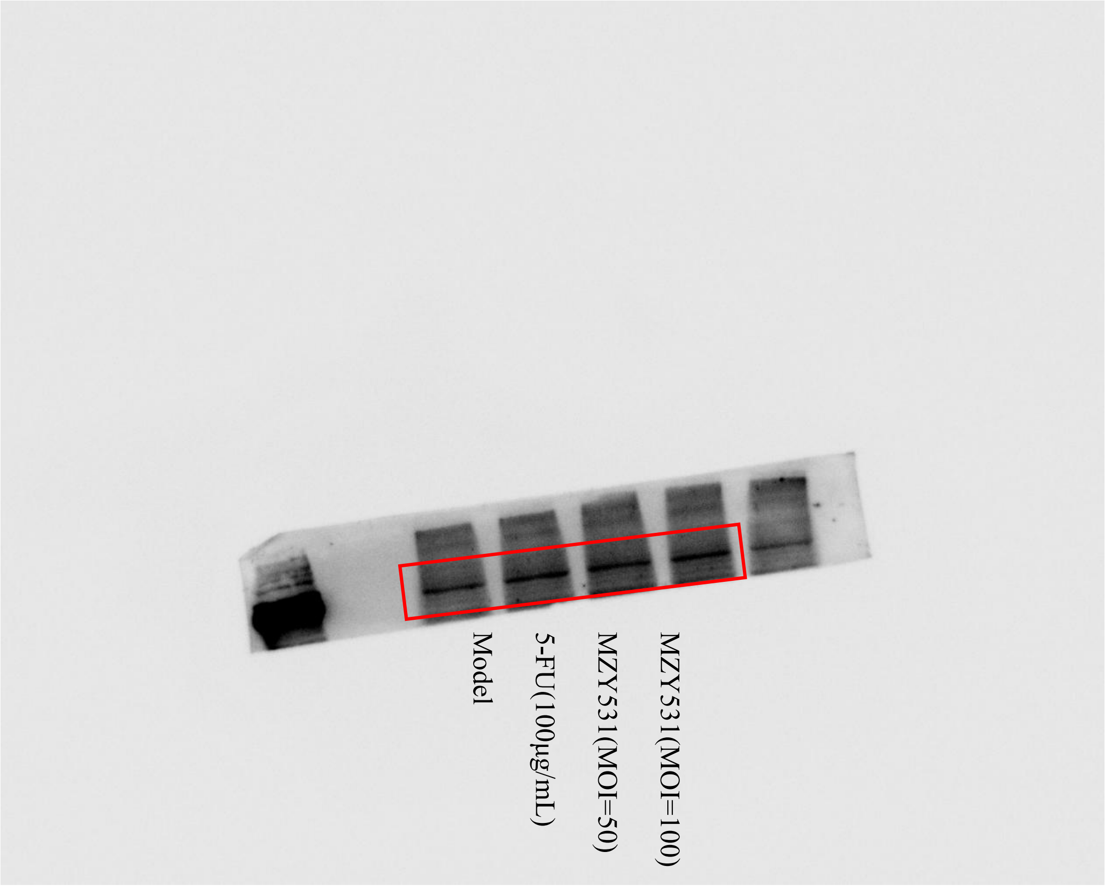

pPI3K-117kDa

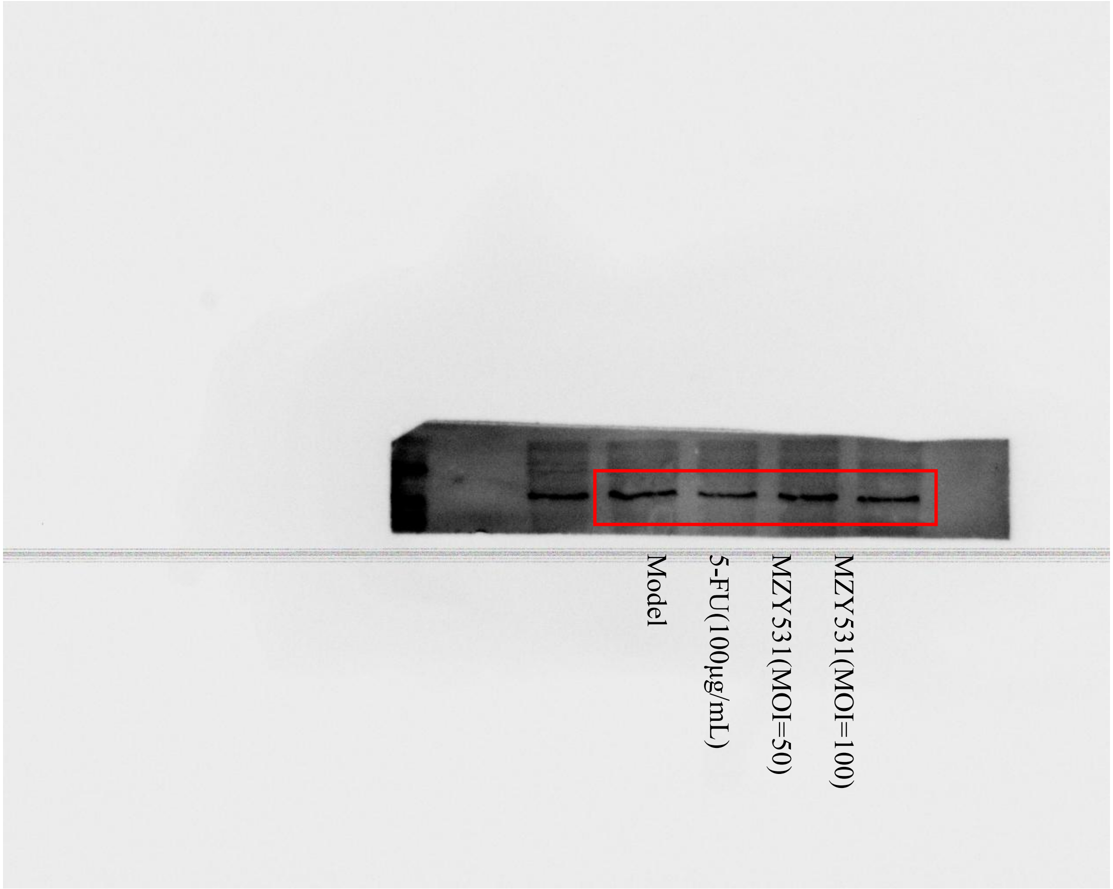

AKT-56kDa

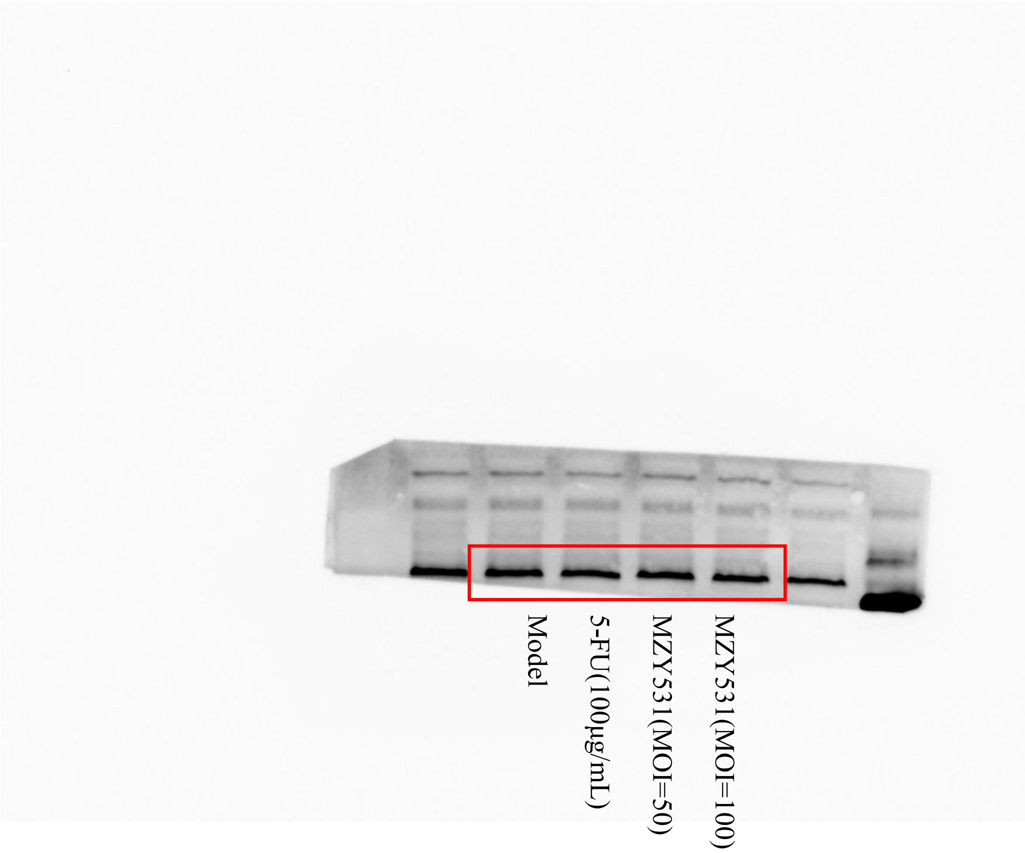

p-AKT-56kDa

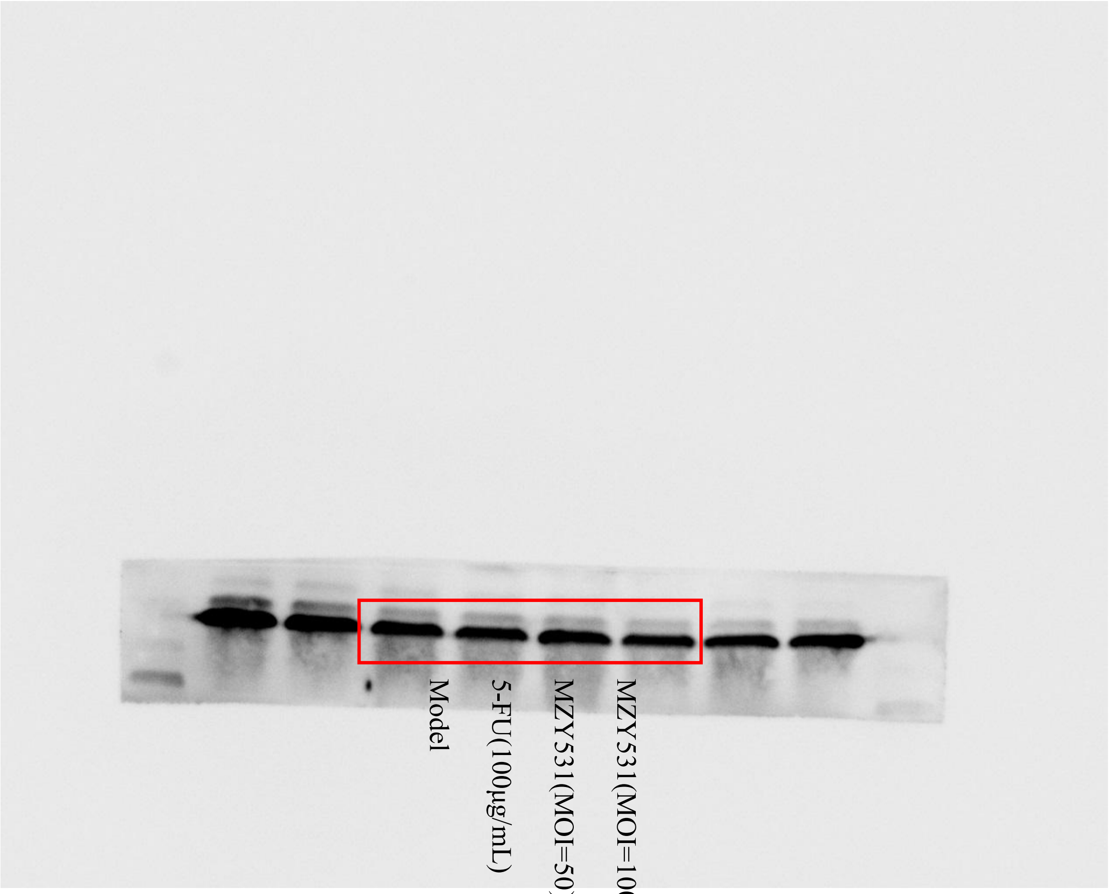

mTOR-289kDa

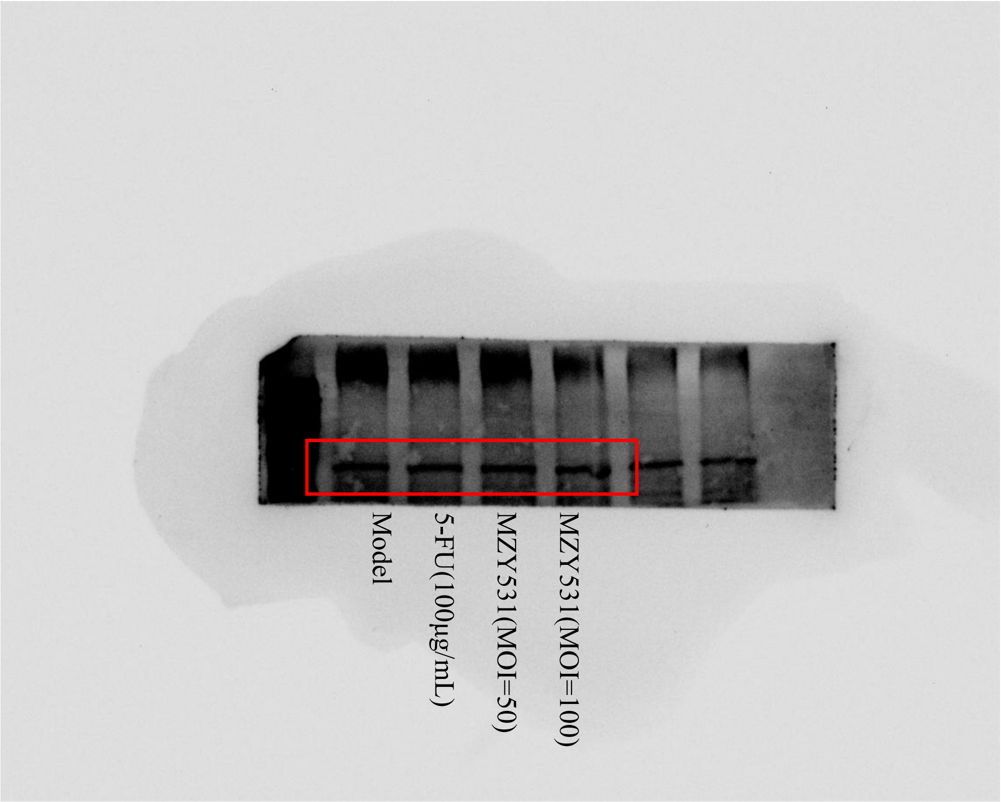

p-mTOR-289kDa

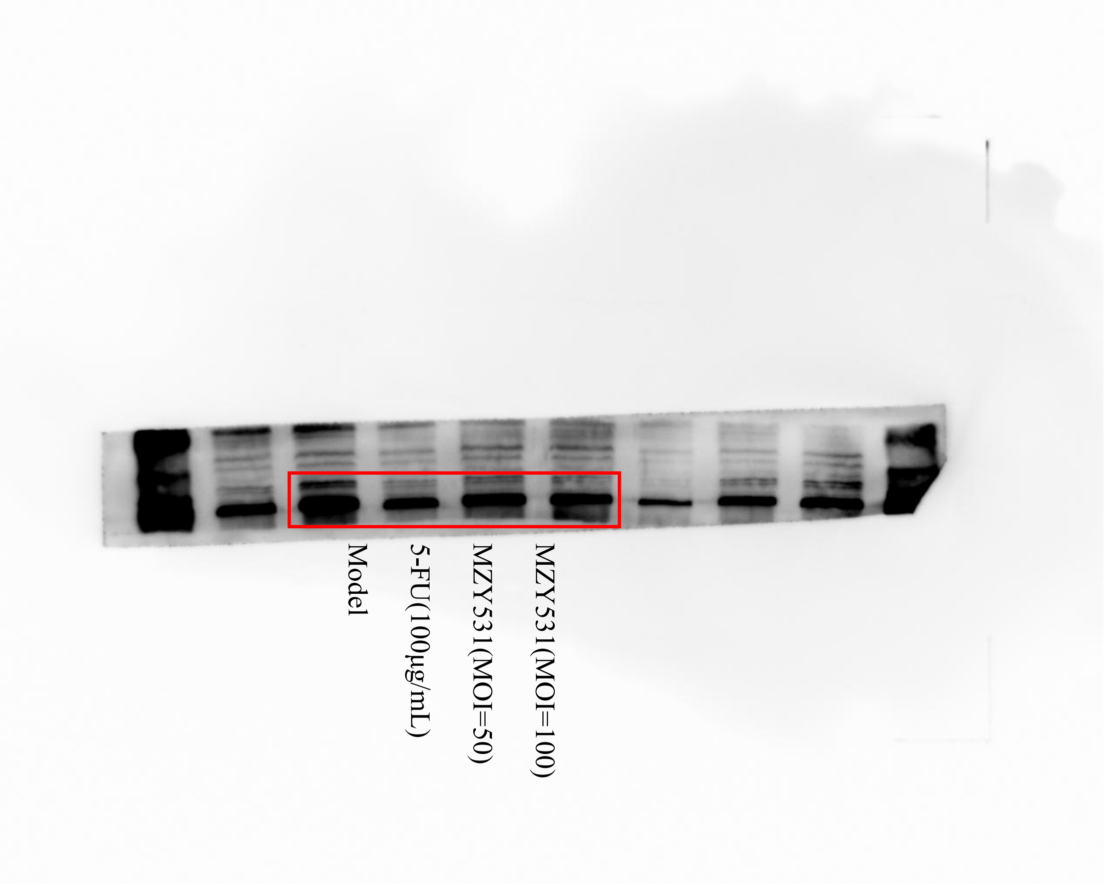

Supplement: Supplementary file 1 — Supplementary Material 1: Raw data of protein bands. [file 12906_2023_4120_MOESM1_ESM.pdf]
